# Measurement and differentiation of banana juice scent using an electronic nose FF-2A

**DOI:** 10.7717/peerj.10638

**Published:** 2021-01-05

**Authors:** Mayumi Nomura, Erika Osada, Tsuyoshi Tokita, Takeo Iwamoto, Yoshinobu Manome

**Affiliations:** Core Research Facilities, The Jikei University School of Medicine, Minato-ku, Tokyo, Japan

**Keywords:** Banana, Juice, Scent, Electronic nose

## Abstract

**Background:**

Banana juice is becoming a popular beverage in Japan and the number of soft-drink stands or shops that take great care and pride in the quality of their products has been increasing. This study aims to measure the scent of banana juice from different brands using the electronic (e-) nose FF-2A in order to identify the characteristics, time-related changes, and the differences among them.

**Methods:**

We standardized the scent value of banana juice measured using FF-2A and determined the absolute value in three different shops. We compared the similarities in samples from each shop with axis data created using standardized measurement. With FF-2A we identified the scent common to all banana juice samples from the composite scent and numerically showed the similarity to the reference gas.

**Results:**

The juices from each shop had their own characteristics and we were able to identify the difference between some of these. The response of FF-2A varied according to the increase/decrease in the number of characteristic molecules measured by GC-MS such as overtime fluctuations in the gas. These data were shown along with the differences between the various banana juices.

**Conclusions:**

FF-2A was able to identify the scent of banana juice at each banana shop as well as time-related changes. By combining GC-MS, we were able to evaluate scent components that changed over time. The results using the electronic nose may prove useful for objective evaluation and comparison of scent with other types of juices.

## Introduction

Various types of fruits are widely served as juice. While apples, strawberries, and grapes are also popular, banana is the most consumed fruits in the United States (Shahbandeh, 2020) and which has advantages over other juices since number of beneficial physiological effects of banana juice have been identified. Treating diabetic rats with freeze-dried banana juice has demonstrated anti-diabetic and anti-hyperlipidemic effects (Dikshit et al., 2012) leading some to suggest health-promoting effects of banana juice in humans. As mentioned above, banana juice is widely consumed throughout the world. This is especially the case in Japan where this particular juice has recently become popular being widely featured in various television programs and magazine articles. Not only is this juice sweet and delicious, but juice also has an excellent scent (Jain & De, 2019) and the number of juice stands specializing in banana juice have been increasing. However, although collectively termed “banana juice”, each shop has its own way of making the juice using bananas of different types and quality.

A sensory test by experts is performed to compare the scent of foods and beverages. However, the sense of smell and taste varies from person to person. This is particularly true for the sense of smell. There are approximately 1,000 odorant receptor protein genes in rodents (Buck & Axel, 1991), whereas in humans there are only about 400 genes to encode functional receptor proteins and just some of these are expressed. Furthermore, the area of the olfactory epithelium is only 10 when that of dog exceeds 170. In addition, signals from the olfactory system are expressed spatially and temporally in the brain (Bear, Connors & Paradiso, 2015); it is therefore difficult for humans to discriminate between them objectively.

On the other hand e-nose can use multiple sensors to comprehensively investigate scent characteristics from its reaction pattern (Persaud & Dodd, 1982). So far we have revealed the differences in coffee aroma using a method which involved the e-nose learning the smell of wine (Fujioka et al., 2015). This method has also been used to distinguish the differences between flower stocks by scent (Fujioka et al., 2012).

Moreover, GC-MS can separate the components in a mixture and quantify the type and amount of the separated components for a mixture of multiple components in a gas that has a high boiling point and carbon as its main component (Poster et al., 2006).

In this study, focusing on the scent of banana juice we aimed to clarify the followings using e-nose FF-2A which is developed as a fragrance recognizer equipped with 10 types of sensors having differing characteristics and GC-MS, whether it is possible to identify the characteristics of the banana juice scent itself and the uniqueness of those characteristics at each shop using a sensor, whether we can tell how the scent will change over time after the juice has been made using the sensor, and whether there is a difference in the scent between different types of banana juice which are currently on sale.

## Material and Methods

### Materials

Banana juice was purchased from three juice stands in Tokyo (shop H, shop O, shop S). Juice shops selling banana juice can be categorized into several types according to their business styles; the three primary types are: (i) specialty shops –that specialize in banana juice, (ii) general beverage stores that assort different types of juice for selling publicly; (iii) and fresh fruit stores that sell juice along with various fruits. In this study we selected one shop representing each of the above types to reduce a potential bias for a specific business style. Of note, some of these shops have several branches; we took samples from those branches that allowed the measurements immediately after the juices were produced. We collected at least three samples from each shop for data verification.

Juice from the three shops was purchased on the morning of the same day, processed in the same way, and measured so that there was no variation between operations. For measuring multiple juices on the same day we ordered each juice separately on one specific day. For examining the variation in juice depending on the day we purchased each juice from different shops on three separate days.

### Measurement of scent of banana juice

Three ml of the purchased juice after a lapse of a specific time was injected into a 2L PET bag (Shimadzu Corporation, Kyoto, Japan) filled with two liters of odorless nitrogen gas. After leaving each PET bag for 15 min at 18 °C, 900 mL of gas sample in the bag was transferred in an air tight manner to another PET bag so that the volatile components from the juice were not mixed. These samples were subjected to measurement for FF-2A and GC-MS.

In order to recognize the scent in a pattern, the resistance value (-log10 (peak/base)) was measured three times by the scent sensor device FF-2A (Shimadzu Corporation, Kyoto, Japan), equipped with ten kinds of oxide semiconductor sensors, all having different characteristics. Details of onboard sensors have not been revealed. These sensors were calibrated and standardized with nine standard gases (hydrogen sulfide, methyl mercaptan, ammonia, trimethylamine, propionic acid, butyraldehyde, butyl acetate, toluene, and heptane) before and after the experiment and measurement was performed at 18 °C under controlled moisture environment. The measured value was extracted (PostPro2 software, Shimadzu Corporation), processed (Asmell2 fragrance analyzing software, Shimadzu Corporation), and nine standard gas vectors based on human odor sensation were built in the space of 10 sensor dimensions. Refer to the literature for the calculation of the similarity to the nine standard gas vectors (Fujioka et al., 2009). Also by measuring the scent of banana juice at each shop while changing the concentration–time in the collection tube (6 s, 18 s, 60 s), we created a new reference axis for each shop virtually and calculated this similarity (Fujioka et al., 2009; Fujioka et al., 2013; Kita et al., 2007). This device has been used in the deodorant test method of ISO-17299 and has been efficaciously recorded for determining complex scent.

FF-2A can obtain data in two modes; the direct mode in which the device measures scent components directly at one measurement protocol, and the capture mode in which the device measures scent components concentrated by introducing heat to the collection tube (Fujioka et al., 2009). The capture mode is suitable for obtaining data from subjects with high boiling point volatility that are difficult to measure as they are. In order to suppress variations in sensor values during analysis, the first and second sensor values which are sometimes affected by the immediately preceding measurement, were excluded from the measurement data. SPSS25 (IBM) was used for statistical analysis. The standardized data obtained were analyzed as non-parametric data and hierarchical cluster analysis was performed using Ward’s method which has a high classification sensitivity as a method of indicating the discriminative power of each sensor and is displayed as a tree diagram (Asakawa et al., 2018).

### Analysis of scent components

In order to know what components are contained in the scent of banana juice, the same gas sample as in FF-2A was used and a gas chromatography mass spectrometer with DB-5MS+DG glass capillary column (Agilent 5975 GC system) was used for the measurement. The molecules were analyzed for components detection by NIST MS Search 2.3.

### Esterase activity measurement

We diluted juice 20 times with double distilled water and 5 µl of sample was immediately subjected to measurement. As the substrate we used 5(6)-carboxyfluorescein diacetate (Marker Gene Technologies, Inc., Eugene, OR, USA) and measured according to the manufacturer’s instructions.

## Results

**Figure 1:**
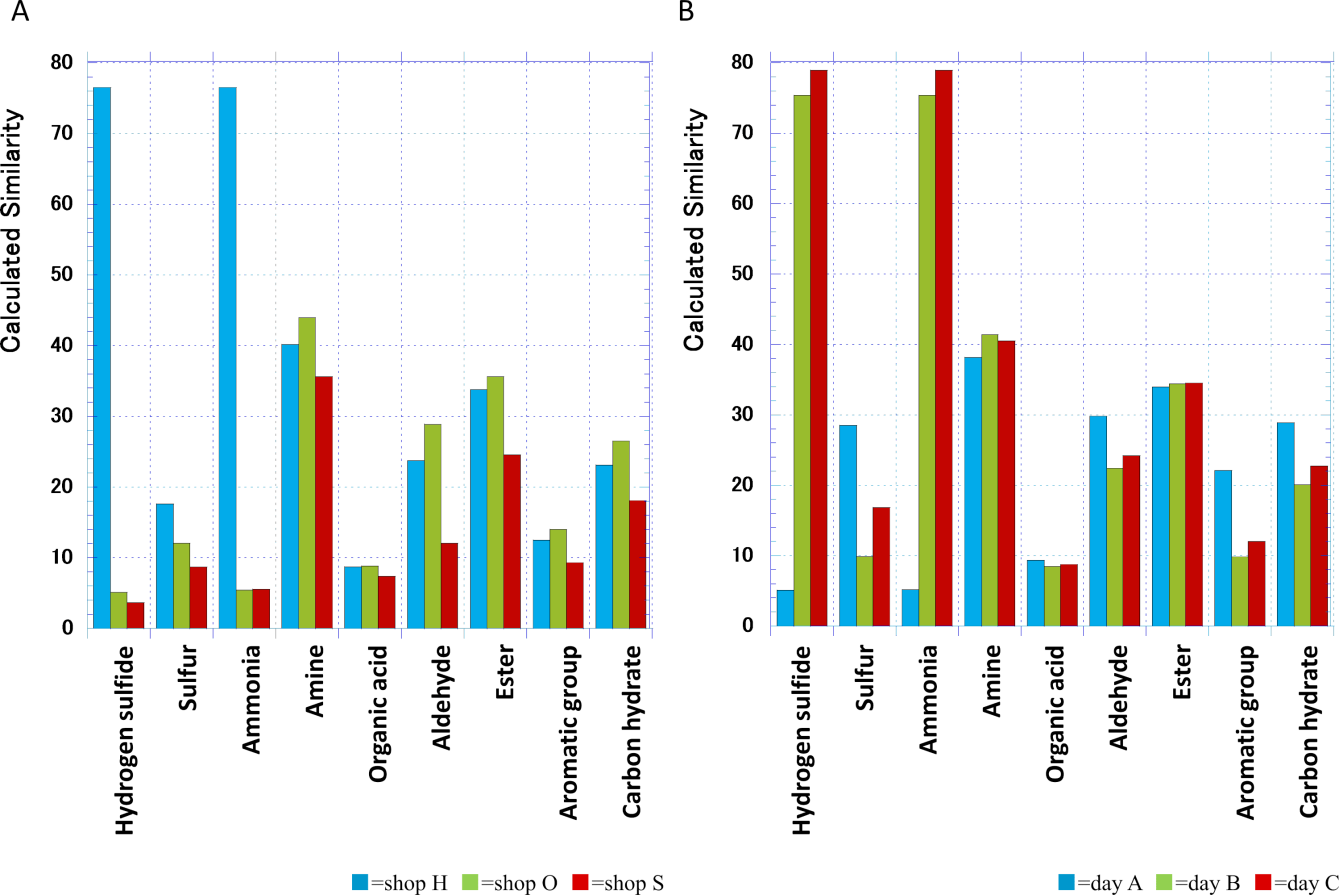
Similarity between banana juice scent and nine different standard gas categories. (A) Comparison between banana juices purchased at three shops. (B) Comparison of the scent of banana juice purchased on different days at shop H.

To examine the type of scent that humans perceive in the banana juice scent, we calculated the similarity to the standard gas vector from the reactivity of the scent of banana juice in three shops to ten types of sensors. As a result the examined banana juice had similarity of more than 20% to hydrogen sulfide, ammonia, amine, ester, aldehyde, and carbon hydrate gas vectors. The similarity to amine, ester, aldehyde, carbon hydrate was common in all shops while similarity to hydrogen sulfide and ammonia were high in only one shop (more than 75%) and low in other shops (less than 10%) (Fig. 1A). Therefore, we hypothesized that the similarity between the hydrogen sulfide axis and the ammonia axis was not due to the similarity of the scent of banana juice itself but to the difference in the conditions for growing bananas and making juice. The advantage of an e-nose is that the values obtained from measuring juice on the same day can be stored on a PC and compared directly with measurements on other days. When we purchased banana juice from shop H three times on another day and compared the juice under the same conditions, it was highly similar to hydrogen sulfide, ammonia, amine, ester, aldehyde, and carbon hydrate values seen in Fig. 1A. In this shop, the axes similar to hydrogen sulfide and ammonia varied greatly from day to day (Fig. 1B). Amine-, ester-, carbon hydrate-, and aldehyde-based scent did not change day to day so we considered these to be the characteristics of banana juice scent.

**Figure 2:**
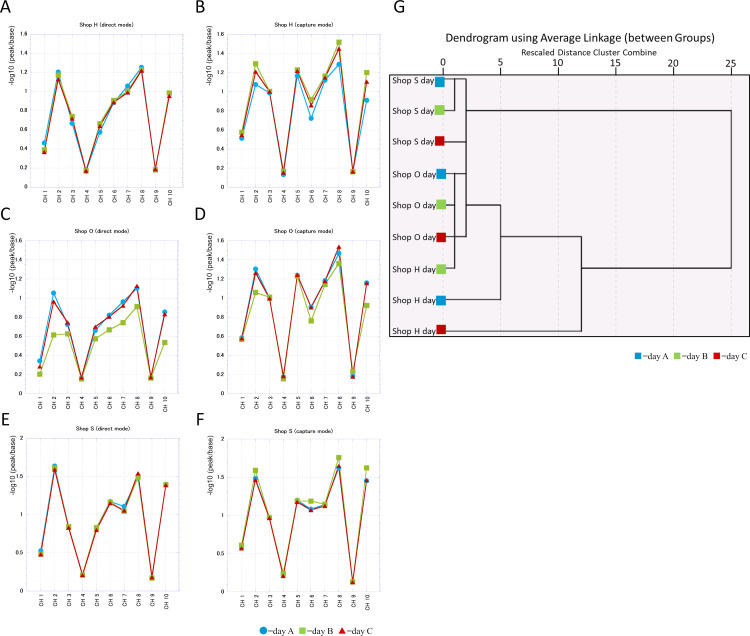
A comparison of the scent of three banana juices purchased at the same time on three different days. (A–F) Reaction of each sensor to the juice scent. (A, C & E) are measured by direct mode; (B, D & F) are measured by capture mode. (G) Dendrogram of standardized sense values in each banana juice obtained from three different days.

We examined the day-to-day differences in each shop by how much the banana juice scent responded to the ten e-nose sensors (Figs. 2A–2F). Each channel (ch) corresponds to each sensor. A, C and E is the measurement by the direct mode; B, D and F is the measurement by the capture mode. FF-2A can perform these two measurements at the same time. Responsiveness to channels 2, 5, 7, 8, and 10 was generally high at all shops and reactivity to 1, 4, and 9 was low. By the direct mode, shop H and shop S showed a small daily difference but shop O had a significant fluctuation depending on the day the banana juice was purchased. Also as for shop H and shop S, we found that there is a tendency for the scent component to differ in the capture mode than direct mode even if the difference is small depending on the day. Banana juice from shop S was the most stable and responded to the sensor in a similar pattern. Shop S chose and froze bananas for use in advance and purchased them in a large lot. This shop put frozen bananas directly into a mixer to make juice at the time of sale. At shop H, bananas were frozen and thawed at the juice stand as well. As for shop O which is a fruit shop, banana juice was not prepared and sold in this manner.

We created a dendrogram from the values measured by both the direct mode and the capture mode and investigated the types of clusters formed by each scent of juice. The banana juices of shop S and shop O formed clusters even on different days and the distances were different from those of banana juice of shop H (Fig. 2G).

**Figure 3:**
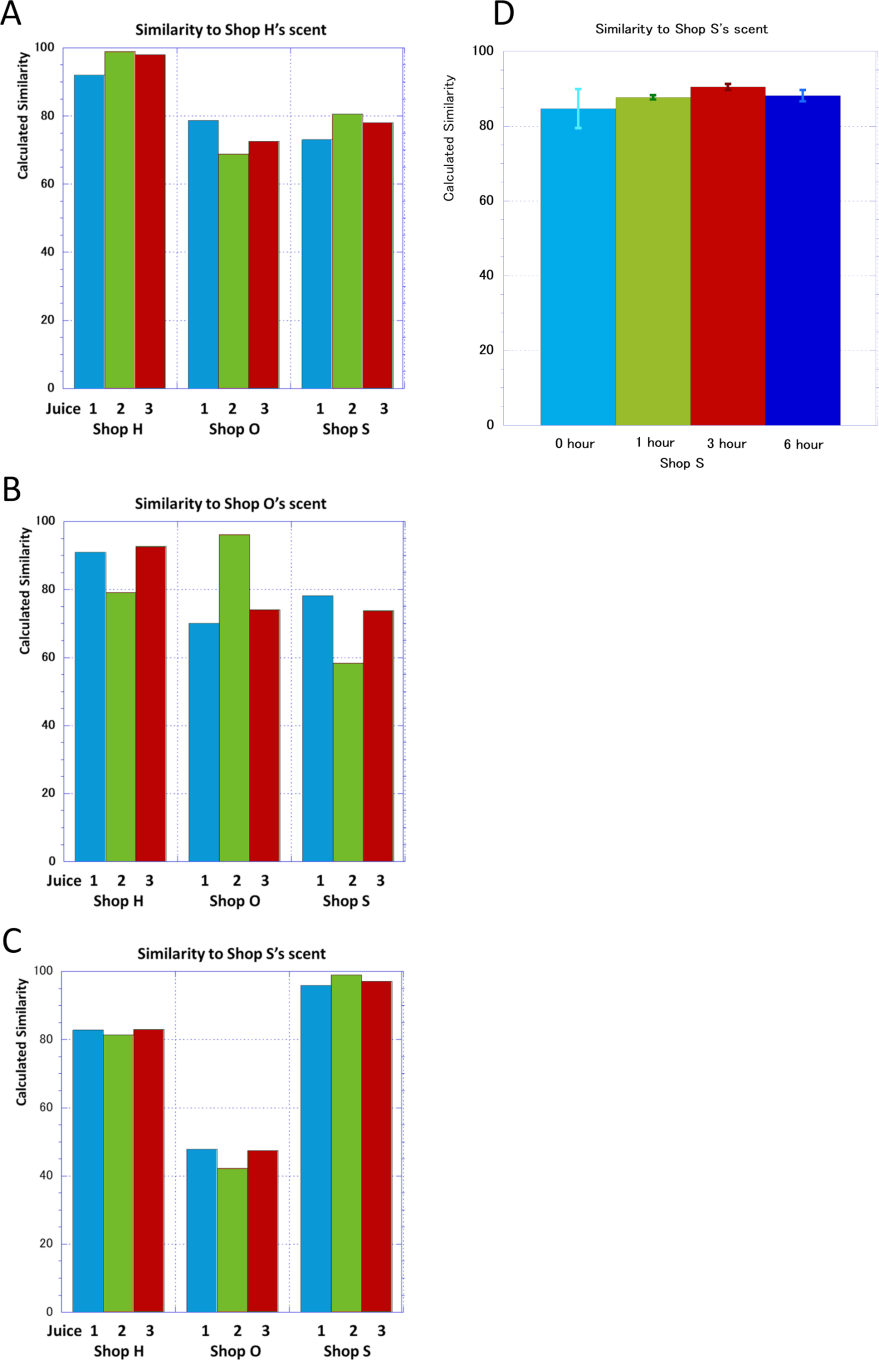
Similarity to the axis with the juice of one day as standard gas at each shop. (A–C) The scent of the three banana juices purchased in each shop and their similarity to the standard gas vector of each shop. (D) Change in the similarity of three banana juices purchased at shop S to standard gas vector of shop S scent over time.

By FF-2A the reference axes can be virtually created from the gas values measured with different concentrations and the similarity can be calculated by projecting the measured values onto those axes. Figures 3A–3C shows the similarity levels in the banana juice of each shop compared on different days using the scent of the shop’s juice as the reference axis in comparison. In shop H the banana juice of any day had the highest similarity to the reference axis, while the results of shop O and S both showed different levels of similarity to a similar extent. Similarity of shop O juices varied significantly on each day. The banana juice from shop S showed a high similarity of 95% or more with the axis of the shop’s banana juice on any day and showed a relatively stable similarity to the scent of shop H. The degree of similarity with the axis was low for shop O.

Banana juice from shop S had a stable scent axis. This shop claims a recommended shelf life of 20 min. Therefore, we purchased three cups of juice independently and examined the time course of the similarity with its juice axis (Fig. 3D). Fresh juice samples of 0 h had difference between them and there was a wide range in the similarities which might be due to lack of characteristics in early stage samples. However, the similarities between samples increased after 1 h and 3 h, and came to close to the juice axis of the shop. However, after 6 h the similarities decreased in comparison to 1 h or 3 h. But even at the 6 hour-time point the similarities were greater than that at 0 h.

**Figure 4:**
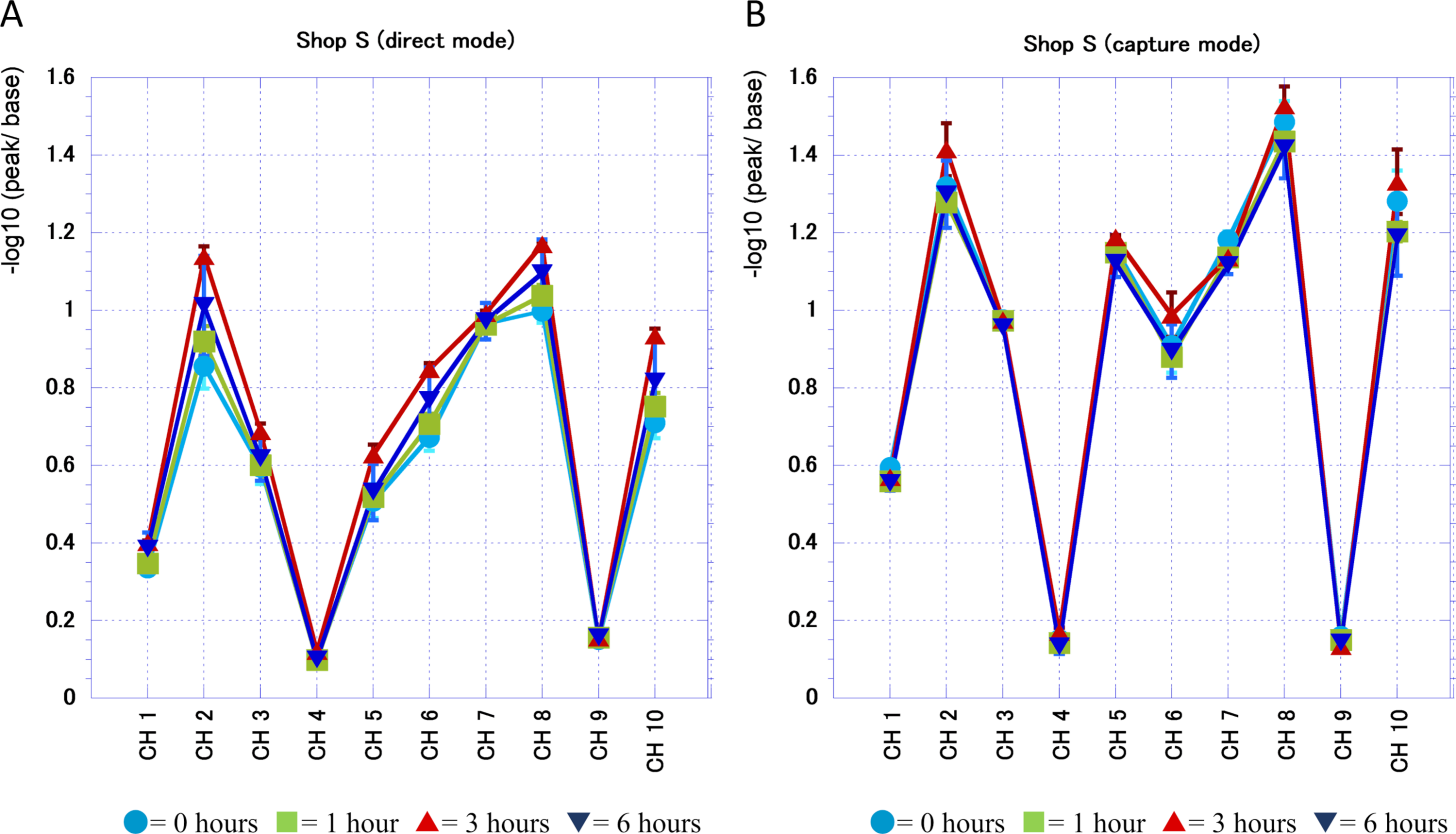
Change of the resistant value of each channel at shop S over time. (A) Direct mode. (B) Capture mode.

After finding that the sensor read and recorded the temporal change in the banana scent, we investigated how each sensor channel captures this change for the banana juice of shop S (Fig. 4). The figure shows an average of three separate banana juice scents examined over time (bars, standard deviation). We detected that the response to the scent of banana juice of the semiconductor sensor differs over time in both the direct mode (Fig. 4A) and the capture mode (Fig. 4B). The sensors of ch4 and ch9 had a weak response to the scent of banana juice and the difference over time was small. By the direct mode some channels, such as ch3 and ch7, showed little change over time but with ch2, ch8, and ch10, the reaction increased after 1 h and 3 h, respectively. However, after 6 h the response to these channels tended to be lower than that after 3 h. Even in capture mode, the response after 3 h was similarly high to ch2, ch8, and ch10. The difference from the direct mode measurement was that the reactivity at 0 h was highest for ch1 and ch7, and that the reactivity to ch5 increased generally. With FF-2A it is difficult to know what kind of scent molecule each channel responded to but these data were useful for later analysis of GC-MS data. The data in Fig. 3D shows the similarity to the axis of the shop. The deviation of the bias was significant at 0 h, but the data in Fig. 4 showed that the reaction with each channel was stable even at 0 h. Therefore, it was considered that the change in the similarity with the axis of the shop was not the difference in the reactivity with each channel but the change to the reference axis.

**Figure 5:**
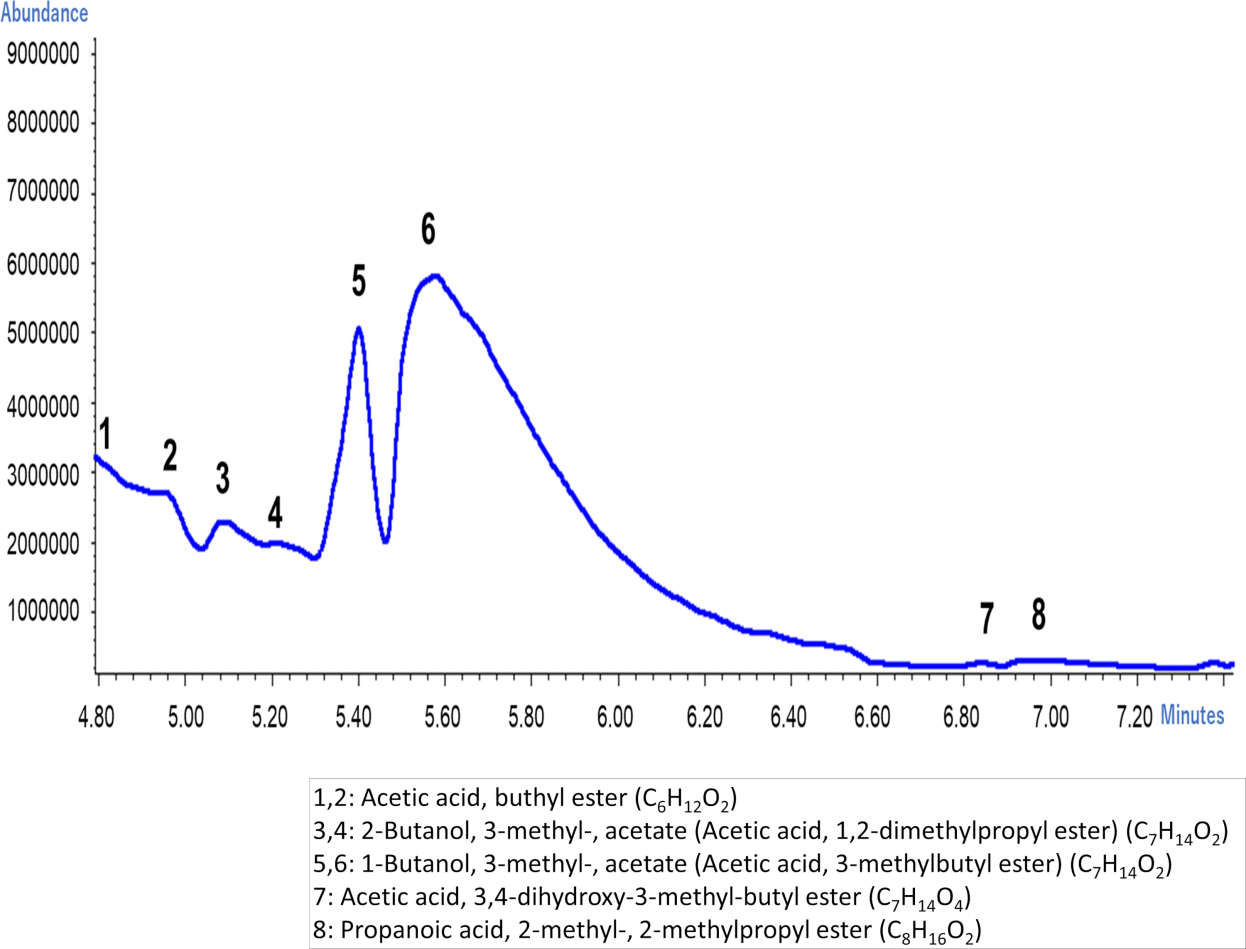
Typical molecules released from banana juice by GC-MS.

We investigated the scent of banana juice of shop S by GC-MS to find out what components were volatilized from the banana juice then several peaks were observed as shown (Fig. 5). Also we investigated the corresponding molecules for each peak and identified the substance as shown in Fig. 5.

**Figure 6:**
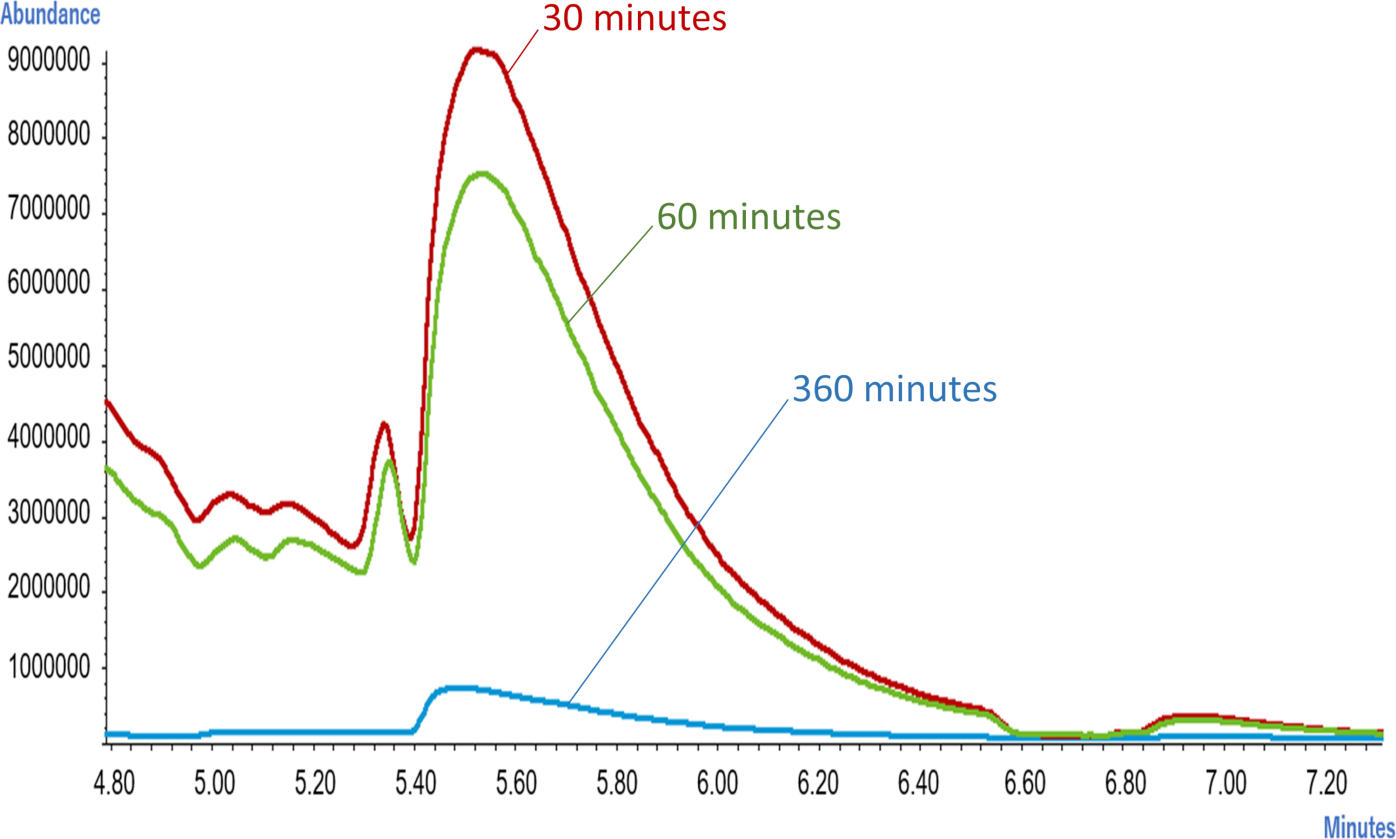
Difference in chromatogram released from banana juice over time (after 30 min).

When the scent of the banana juice of shop S was evaluated with a sensory test, change of intensity of scent in 30 min, 60 min, and 360 min was not recognizable. However, the aroma characteristics changed from Floral (honeysuckle) and Stone fruit (peach, apricot) to an aroma associated with Tropical fruit (mango, pineapple) after 60 min. We investigated peak fluctuation with GC-MS and found that the 1-Buthanol, 3-methyl-, acetate corresponds to peaks 5 and 6 in Fig. 5 at the high peaks of 30 min (Fig. 6). We realized that the peaks fell drastically after 60 min. The molecule showing these peaks are also known as banana oil. After 360 min the peak fell at almost all retention times. This was contrastive to the data presented by e-nose (Fig. 4).

**Figure 7:**
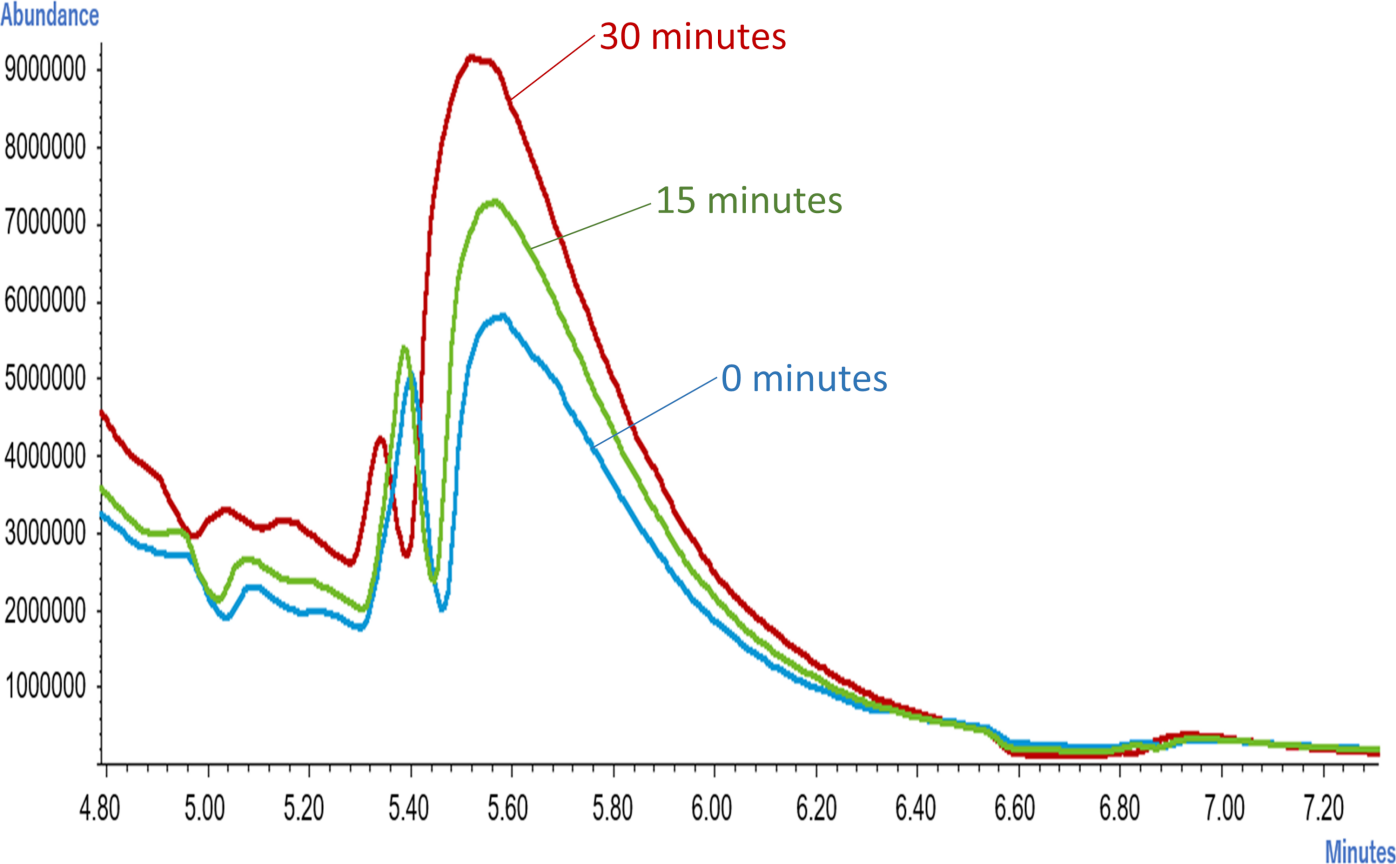
Difference in chromatogram released from banana juice over time (up to 30 min).

We theorized that the decrease of the peak might be related to the deterioration of banana scent, so we analyzed the scent of banana juice immediately after purchase to examine the temporal change (Fig. 7). It was found that the peak was relatively low at 0 min after the juice was made, and corresponding molecules were released into the scent of banana juice after 15 min. In other words the decrease of the peak value after 30 min in Fig. 6 does not indicate that the substances that were already present in the scent of the juice continue to fall, but that the substances are emitted from the banana juice after 15 min. None of the authors noticed the change in the intensity of the scent from 0 to 30 min of the banana juice and the substance alone did not correlate with specific sensory channels of e-nose.

**Figure 8:**
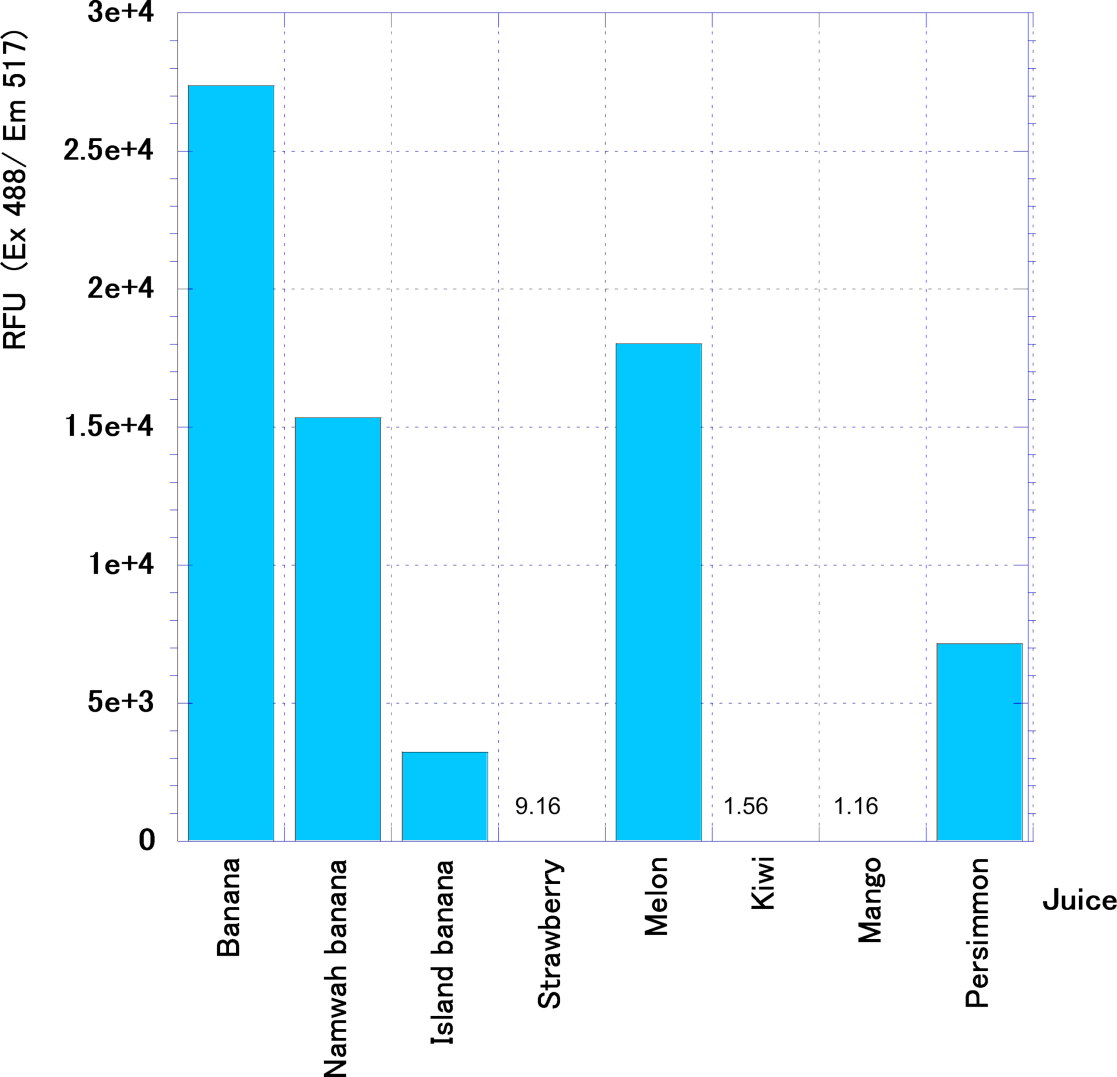
Esterase activity in typical fruit juices. Activities are demonstrated by relative fluorescence units (RFU).

According to the results of GC-MS, the banana scent contained many esters. Since the ester component contained in the scent of banana juice decreased after 30 min, we measured how much ester degrading enzyme was contained in banana juice (Fig. 8). Banana juice was shown to contain higher esterase activity than other juices when we compared to typical fruit juices sold at juice stands. The next highest was melon juice. Almost no esterase activity was detected in strawberry juice, kiwi juice, or mango juice. Even for the same banana there are several bananas including Namwah (Awak) and Island (product of Okinawa, Ogasawara variety) banana. These were sold as premium juices at this shop. When these juices were compared with regular banana juice, Namwah banana juice yet had esterase activity but Island banana activity was less than 1/8.

**Figure 9:**
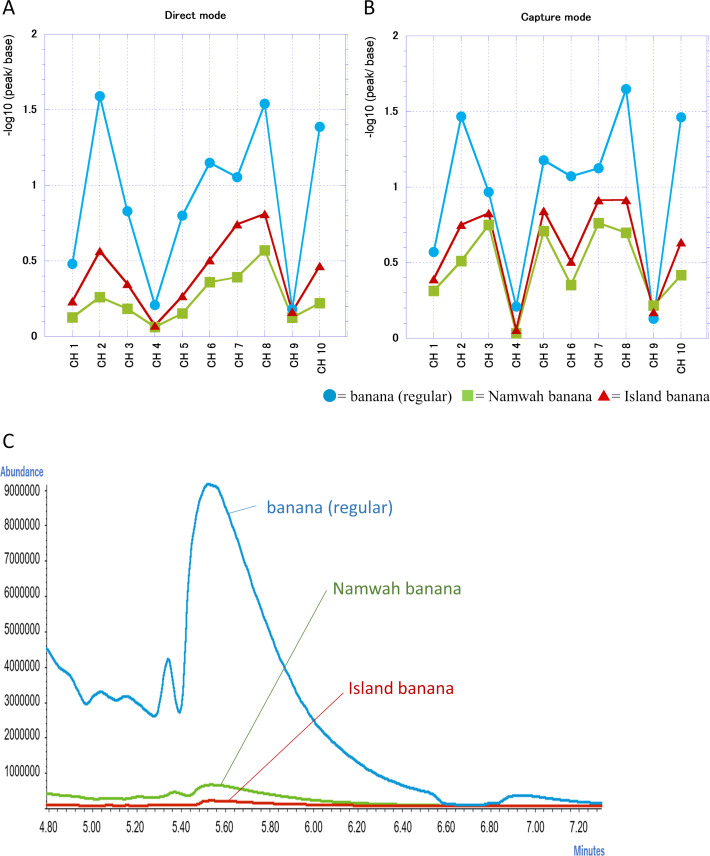
Comparison of shop S regular bananas with Island banana and Namwah banana juice. (A–B) Change of the resistant value in each channel of FF-2A. (A) Direct mode, (B) Capture mode. (C) Chromatogram comparison.

We compared the scent of juices from regular banana, Namwah banana and Island banana of after 60 min by e-nose (Figs. 9A and 9B) and GC-MS (Fig. 9C). Namwah bananas and Island bananas demonstrated a difference from regular banana juice in their reaction at almost all channels by FF-2A.

## Discussion

The e-nose senses the scent using a sensor and records the reaction with the sensor as a pattern just like humans do. Thereby it replicates the conventional sensory evaluation of scent by humans. For example, FF-2A is equipped with an algorithm which can digitize and pattern the scent by comparing it with a preset reference gas as well as describe the scent quality (similarity to the reference gas) and strength (scent index equivalent value) of the sample as absolute values (Shimadzu, 2020). This method can standardize the scent recorded as an absolute value; thus, it can be used to evaluate the recorded scent. We have reported a method to set a cut-off value objectively by extracting characteristics of apple scent in comparison with scent patterns of various fruits to combine it with analysis using a Receiver Operating Characteristic (ROC) curve (Asakawa et al., 2018).

Various kinds of e-nose systems have been applied to banana and used to verify its quality (Sanaeifar et al., 2016), aging (Llobet et al., 1999; Sanaeifar et al., 2014a) sorting (Sanaeifar et al., 2014b), and so on. On the other hand, another report also argues that the ingredients of banana juice are essential for examining the maturity and quality of juice (Baietto & Wilson, 2015). The presence of this particular report was encouraging for our study. However, we should note that the results obtained from e-noses are device-dependent; we must therefore take sensor characteristics of each result when applying them to FF-2A.

Humans identify scent using the olfactory center of the brain through pattern analysis of information obtained from olfactory receptor cells responding to the characteristics and concentrations of scent ligands of odorants (Shepherd, 2006). On the contrary, the e-nose sensors demonstrate differences in responsiveness to scent depending on differences in sensor specifications; they demonstrate differences in the output pattern depending on differences in sensor reaction to scent molecules.

As mentioned above, the human olfactory center and the scent sensor device function similarly to each other in terms of scent identification through pattern analysis; the scent sensor device is thus useful for quick analysis of scent characteristics. In fact, some research projects are currently in progress to identify food quality and detect diseases using scent sensors (Loutfi et al., 2015; Turner & Magan, 2004). However, human olfactory receptors are 7-transmembrane G protein-coupled receptors which show different reactions from those of scent sensors. When a scent molecule binds to a receptor, G protein activates adenylate cyclase to convert ATP to cyclic AMP (cAMP); then cAMP opens an ion channel and sodium ions enter the cell to induce cell depolarization to send action potentials to the brain (Zufall, Firestein & Shepherd, 1994). In other words, the human scent-recognition mechanism has a scent threshold value and electrical signals are generated on an all-or-none basis to be transmitted to the nerve axon; this mechanism is fundamentally different from that of a sensor device which identifies scent molecules in a dose-dependent manner.

The GC-MS could identify the types, extent, and timing of molecule being released. So far, more than 150 banana volatiles are known (Shiota, 1993). It was not the goal of this study to analyze individual volatile components; thus only representative peaks are presented here. However, it should be noted that esters were included in the components whose value changed over time after the preparation of the juice.

Studies on biosynthetic pathways of volatile constituents in fruits have become more advanced and it has long been known that ester formation is associated with banana ripening (Jayanty et al., 2002); however, it is also well known that bananas contain esterase and the varieties of banana can be classified by their isozymes (Dutta et al., 2003; Mandal et al., 2001). Our data also showed that banana juice had higher esterase activity than other typical fruit juices. Some vegetables such as pumpkin and eggplant which do not demonstrate ester formation have high esterase activity, and there is no correlation between the strength of esterase activity and ester formation. Also, an examination of the changes in esterase activity and ester-forming ability of bananas should demonstrate that esterase does not produce ester by its reverse reaction. Regarding banana flesh, no ester formation was observed in ripe green fruits. However, ester activity increased in yellow-green fruits and peaked in yellow-ripened fruits and dark-yellow ripe fruits. Ester-forming ability also decreased in overripe fruits. The esterase activity shows an increasing trend with the progress of ripening, but the period of highest esterase activity does not coincide with the period of ester formation. The esterase activity is present even in ripe green fruits and the highest activity is seen immediately after the stage of overripe fruit (Ueda & Ogata, 1977).

In our results we found that the ester scent in banana juice rises soon after the mixer crushes the banana and the ester in the banana evaporates. Despite this result, it is quite unlikely that the ester content decrease in the banana scent after 30 min is solely associated with the phenomenon of comprehensive evaporation of the scent component in the juice. It was also reported that the addition of casein increased the esterase activity in bananas (Ueda & Ogata, 1977) and esterase activity in banana juice may play a part in the disappearance of ester content when banana juice is mixed with milk. Further examination is necessary in this regard.

In addition to regular banana juice, shop S sells Island banana juice and Namwah banana juice as premium products. In bananas the volatile components change depending on variety but varietal differences in the volatile profiles are primarily quantitative, and only a few compounds are variety-specific (Baietto & Wilson, 2015). Therefore, it seems unlikely that the juice has its own volatile constituents.

We demonstrated that the detected reactivity with scent and its components was less than that of regular banana juice by both e-nose and GC-MS. However, there was a discrepancy between the e-nose and GC-MS results for the two banana juice scents. The peak for the volatile components of the Island banana was exceptionally low in GC-MS (lower than Namwah banana in Fig. 9C) while Namwah banana showed a slightly less reactivity than Island banana in each channel for the e-nose response (Figs. 9A and 9B). Generally semiconductor sensors equipped in e-nose have a very high sensitivity (the sensor used in this study can detect up to 0.05 ppb) and no matter how many molecules appear in the gas, they cannot be detected unless they react with the sensors. This is a point to be considered when comparing e-nose data with GC-MS data. This led us to the results showing that, even in the data obtained 360 min after the preparation of banana juice, the peaks of many components were significantly decreased in GC-MS compared with those in 60 min but the response to e-nose did not decrease as much.

The generalization of this study is as follows: although characterizing the types of molecules and assessing their amounts using GC-MS as well as evaluating their sensor reactivity with the e-nose are both different from sensory evaluations based on actual memories in the brain, these approaches allow the recording of scent properties quantitatively; thus a direct comparison can be performed even when the number of samples is small. Therefore, the use of e-nose provides a significant advantage since it allows the recording of scent properties as absolute values.

On the other hand this study was mainly limited by the requirement of products that maintained a stable scent axis for properly analyzing samples from shops. Even when samples suitable for proper comparison are available from the shops, we need to consider that the lot of stocked bananas will change as will the respective scent axis. When this happens, we can nevertheless compare how the scent properties change as numerical values by creating a new scent axis.

Finally the evaluation of juice is related not only to the scent but also to taste and appearance. Namwah bananas and Island bananas had a lower scent intensity than ordinary bananas but we considered delicious after drinking their juices. In the human olfactory center the olfactory stimulus enters the amygdala from an olfactory nerve and is processed as information in the diencephalon and cerebral hemisphere which are mostly located in the upper part of the brain. On the other hand, taste is sensed by taste buds and enters the lower part of the brain from the nucleus of the solitary tract of the medulla oblongata via the intermediate nerve in the facial nerve. It then follows an entirely different route in which information is absorbed and processed in the brain stem (Bradley & Grabauskas, 1998). Unlike potato juice and other fruit juices, the anti-browning effect of polyphenol oxidase by beta-cyclodextrin and so forth are not suppressed in banana (Singh, Jadhav & Singhal, 2015). In fact the color of banana juice gradually turned to brown over time, and after 180 min or more, its visual appeal was entirely lost with the juice appearing unappetizing. In that sense it should be necessary to develop a comprehensive evaluation method for making properly integrated judgments of banana juice by using not only olfactory sensors but also taste sensors as well as with visual inspection of its appearance changes using cameras.

## Conclusions

By measuring the scent of banana juice for each of the three shops, each day, and each of the designated time points, and performing absolute value conversion with FF-2A, we were able to record the characteristics of each banana shop and their changes over time. Also by combining GC-MS we could analyze which scent components are changing in juice, and measure the changes in these scent components over time. Future inclusion of evaluation by taste and vision in addition to scent, may be useful for objective evaluation and comparison of the juice scent or others.

##  Supplemental Information

10.7717/peerj.10638/supp-1 Supplemental Information 1 Data from FF-2A.

## Additional Information and Declarations

### Data Availability

The following information was supplied regarding data availability: Raw data are available in the Supplemental Files.
